# In-hospital antibiotic use for severe chronic obstructive pulmonary disease exacerbations: a retrospective observational study

**DOI:** 10.1186/s12890-023-02426-3

**Published:** 2023-04-25

**Authors:** Anna Vanoverschelde, Chloë Van Hoey, Franky Buyle, Nadia Den Blauwen, Pieter Depuydt, Eva Van Braeckel, Lies Lahousse

**Affiliations:** 1 Department of Bioanalysis, Pharmaceutical Care Unit, Ghent University, Ghent, Belgium; 2 Department of Pharmacy, Ghent University Hospital, Ghent, Belgium; 3 Medical Coding Department, Ghent University Hospital, Ghent, Belgium; 4 Department of Internal Medicine and Paediatrics, Faculty of Medicine and Health Sciences, Ghent University, Ghent, Belgium; 5 Department of Intensive Care, Ghent University Hospital, Ghent, Belgium; 6 Department of Respiratory Medicine, Ghent University Hospital, Ghent, Belgium

**Keywords:** Chronic obstructive pulmonary disease, Severe exacerbations, Antibiotics

## Abstract

**Background:**

The use of antibiotics in mild to severe acute exacerbations of chronic obstructive pulmonary disease (COPD) remains controversial.

**Aim:**

To explore in-hospital antibiotic use in severe acute exacerbations of COPD (AECOPD), to analyze determinants of in-hospital antibiotic use, and to investigate its association with hospital length of stay (LOS) and in-hospital mortality.

**Methods:**

A retrospective, observational study was conducted in Ghent University Hospital. Severe AECOPD were defined as hospitalizations for AECOPD (ICD-10 J44.0 and J44.1) discharged between 2016 and 2021. Patients with a concomitant diagnosis of pneumonia or ‘pure’ asthma were excluded. An alluvial plot was used to describe antibiotic treatment patterns. Logistic regression analyses identified determinants of in-hospital antibiotic use. Cox proportional hazards regression analyses were used to compare time to discharge alive and time to in-hospital death between antibiotic-treated and non-antibiotic-treated AECOPD patients.

**Results:**

In total, 431 AECOPD patients (mean age 70 years, 63% males) were included. More than two-thirds (68%) of patients were treated with antibiotics, mainly amoxicillin-clavulanic acid. In multivariable analysis, several patient-related variables (age, body mass index (BMI), cancer), treatment-related variables (maintenance azithromycin, theophylline), clinical variables (sputum volume and body temperature) and laboratory results (C-reactive protein (CRP) levels) were associated with in-hospital antibiotic use independent of sputum purulence, neutrophil counts, inhaled corticosteroids and intensive care unit of which CRP level was the strongest determinant. The median hospital LOS was significantly longer in antibiotic-treated patients (6 days [4–10]) compared to non-antibiotic-treated patients (4 days [2–7]) (*p* < 0.001, Log rank test). This was indicated by a reduced probability of hospital discharge even after adjustment for age, sputum purulence, BMI, in-hospital systemic corticosteroid use and forced expiratory volume in one second (FEV_1_) (adjusted hazard ratio 0.60; 95% CI 0.43; 0.84). In-hospital antibiotic use was not significantly associated with in-hospital mortality.

**Conclusions:**

In this observational study in a Belgian tertiary hospital, in-hospital antibiotic use among patients with severe AECOPD was determined by the symptom severity of the exacerbation and the underlying COPD severity as recommended by the guidelines, but also by patient-related variables. Moreover, in-hospital antibiotic use was associated with a longer hospital stay, which may be linked to their disease severity, slower response to treatment or 'harm' due to antibiotics.

**Trial registration:**

Number: B670201939030; date of registration: March 5, 2019.

**Supplementary Information:**

The online version contains supplementary material available at 10.1186/s12890-023-02426-3.

## Background

Chronic obstructive pulmonary disease (COPD) is a common lung disease characterized by persistent respiratory symptoms and airflow limitation [1]. A major contributor to the overall severity of COPD and the associated burden on health-care systems worldwide, is acute exacerbations of COPD (AECOPD) [1, 2]. AECOPD are episodes of worsening of respiratory symptoms, leading to a change in medication (mild-moderate) or hospitalization (severe) [1, 2]. Exacerbations arise as a result of environmental triggers and/or respiratory tract infections, encompassing viral and bacterial infections [3]. A meta-analysis including 118 studies demonstrated that half of the infections in AECOPD patients are bacterial [4], and might require active antibiotic treatment [5]. However, the use of antibiotics in mild to severe exacerbations remains controversial [6]. Pooled results of four randomized controlled trials in a 2018 Cochrane meta-analysis did not show that the currently used antibiotics significantly reduce the risk of treatment failure, duration of hospital admission or mortality in hospitalized patients, excluding patients in intensive care [6]. Moreover, any antibiotic use, particularly unnecessary or inappropriate use, contributes to the selection of resistant pathogens [7].

The conflicting evidence called for research in biomarkers which could help in selecting patients who benefit from antibiotic treatment to limit overuse. One of the most frequently discussed markers that can be used to guide antibiotic therapy is sputum purulence [6]. The Global Initiative for Chronic Obstructive Lung Disease (GOLD) guidelines recommend antibiotic treatment if patients have at least two of three cardinal symptoms, including increased sputum purulence [1]. This recommendation is based on the results of the historical randomized crossover trial by Anthonisen et al*.* [8], and is supported by a more recent meta-analysis showing a moderate level of evidence that purulent sputum during AECOPD increased the probability of potentially pathogenic bacteria by two-fold [9]. In Belgium, the Infectiology Guide from the Belgian Society for Infectiology and Clinical Microbiology recommends considering the underlying COPD severity in addition to the Anthonisen criteria [10]. Other studies have indicated the use of C-reactive protein (CRP) to guide antibiotic therapy [11]. A randomized controlled trial including patients hospitalized with an AECOPD reported a significantly lower antibiotic use in the CRP-guided (cut-off ≥ 50 mg/L) group (31.7%) than in the GOLD-guided (increased sputum purulence in combination with dyspnea and/or sputum volume) group (46.2%), without an increase in adverse events [12]. CRP-guided treatment is implemented in the Belgian outpatient guidelines due to a more recent revision in 2021 [13], but not yet in the Belgian inpatient [10], and GOLD guidelines [1].

In addition to the decision to initiate antibiotic therapy, the choice of an appropriate antibiotic is of great importance [7]. According to the GOLD 2022 guidelines, the initial empirical therapy could be an aminopenicillin with clavulanic acid, a macrolide or tetracycline, based on the local bacterial resistance pattern [1]. However, GOLD warns for the presence of gram-negative bacterial infections in patients with frequent exacerbations, severe airflow limitation and/or need for mechanical ventilation [1]. The antimicrobial drug recommended by the Belgian Infectiology Guide based on national resistance is amoxicillin-clavulanic acid, but another antibiotic is preferred in case of immunoglobulin E (IgE) mediated penicillin allergy (moxifloxacin) or risk factors for *Pseudomonas aeruginosa* infection (anti-pseudomonal antibiotic such as piperacillin-tazobactam). The total recommended duration of antibiotic therapy was 5 days and rotation of antibiotics was not recommended [10].

To the best of our knowledge, studies that investigated the determinants of antibiotic use for AECOPD were performed in primary care [14, 15], or did not investigate laboratory results such as CRP [16]. Only two retrospective studies investigated the real-life effect of (guideline-recommended) antibiotics in patients hospitalized with AECOPD on outcomes, both performed in the United States [17, 18]. Since data on antibiotic use of severe AECOPD in Belgium are lacking, we aimed to describe the antibiotic use including initial choice of antibiotic in severe AECOPD patients, to analyze determinants of in-hospital antibiotic use, and to investigate its associations with important outcomes such as length of stay (LOS) and in-hospital mortality.

## Methods

### Study design

Antibiotic use for treatment of severe AECOPD was investigated in a retrospective, observational study in Ghent University Hospital, a Belgian tertiary hospital with more than 1000 beds.

### Data sources

This study was conducted using three data sources. First, the Minimal Hospital Data (MHD) registration database from Ghent University Hospital was used to select hospitalizations for AECOPD. Additionally, patient characteristics were collected from the electronic patient records (EPR). Finally, the use of systemic antibiotics and corticosteroids was collected from the hospital pharmacy data.

### Study population

Inpatients above the age of 40 hospitalized in regular ward or the intensive care unit (ICU), who were discharged between January (Jan.) 1^st^, 2016, and Jan. 1^st^, 2022, and who received an International Classification of Diseases 10^th^ Revision (ICD-10) code for an AECOPD present on hospital admission, were included in this study. The selection of the ICD-10 codes from the ICD-10 codebook consists of COPD with acute lower respiratory infection (J44.0) or COPD with (acute) exacerbation (J44.1) as verified admission diagnosis. These codes are co-determining in the assignment of the All Patient Refined Diagnosis Related Groups (APR-DRG) 140 (COPD) through the 3 M APR-DRG algorithm. Patients with a concomitant diagnosis of pneumonia or ‘pure’ asthma were excluded based on the ICD-10 codes (J13-J18 and J45, respectively) or diagnosis in EPR. We only analyzed the index hospitalization, defined as the patient’s first hospitalization during the study period to exclude related events.

### Assessment and analysis of drug use

Information was retrieved using the Anatomical Therapeutic Chemical (ATC) codes for antibacterials for systemic use (J01) and corticosteroids for systemic use (H02). Antibiotic-treated patients were defined as persons with at least one dispensing of an antibacterial for systemic use during hospitalization, independent of use before the hospitalization. Prevalence of in-hospital antibiotic use was defined as the number of antibiotic-treated patients divided by the number of patients hospitalized for an AECOPD.

Antibiotic treatment patterns were captured at the ATC 5^th^ level (chemical substance) and categorized into the mainly used antibiotics: azithromycin with/without another antibiotic on the same day (J01FA10 w/wo other), amoxicillin-clavulanic acid (J01CR02 w/wo other), piperacillin-tazobactam (J01CR05 w/wo other), and moxifloxacin (J01MA14 w/wo other). If more than one of the previously mentioned antibiotics was dispensed on the same day, it was classified under the antibiotic with the highest antibiotic spectrum index [19]. Other antibiotics were categorized as “other”. Antibiotic treatment patterns from admission (= day 0) to discharge or until day 10 were visualized using an alluvial plot generated using R 1.4. The number of dispensed antibiotic units was converted into defined daily doses adjusted to the Belgian situation in hospitals (DDAs, version 2019). A proposal of DDAs was made by Sciensano based on the Belgian Infectiology Guide and a validation was performed by the Flemish Association of Hospital Pharmacists working group on antibiotics and the Belgian Antibiotic Policy Coordination Committee (BAPCOC) working group Hospital Medicine. Median DDAs per hospitalization were used to estimate the days of antibiotic treatment.

### Outcomes

Outcomes were hospital LOS and in-hospital mortality. Hospital LOS was defined as the number of days between the hospital admission date and the hospital discharge date. In-hospital mortality was defined as death occurring during hospital stay.

### Covariables assessment

Covariables were classified as 1) patient-related variables, 2) hospitalization-related variables, 3) clinical variables, 4) treatment-related variables, and 5) laboratory test results.

Patient-related variables were extracted from the MHD (age on admission and sex) or collected from the EPR: height (centimeters) and weight (kilograms) to calculate the body mass index (BMI, kg/m^2^). Frequent comorbidities were classified using the Elixhauser Comorbidity Software Refined for ICD-10-CM Reference File, v2022.1 (Agency for Healthcare Research and Quality).

Hospitalization-related variables were extracted from the MHD. The hospital discharge dates were categorized into winter months from Jan. to March and October (Oct.) to December (Dec.), and summer months from April to June and July to September (Sept.).

Clinical variables were collected from the EPR. Stable (most recent before hospitalization) forced expiratory volume in one second (FEV_1_) was used to categorize the patients into GOLD stage I (≥ 80%), II (50–79%), III (30–49%) or IV (< 30%) [1], and supplemented with GOLD stage written in the EPR if no spirometry was available before hospital admission. The Anthonisen criteria on admission (increased dyspnea, increased sputum volume or purulence) were collected, and the exacerbations were categorized into type 1 (all three cardinal symptoms), type 2 (two cardinal symptoms) and type 3 (one cardinal symptom) [8]. Body temperature was defined as the highest body temperature within 48 h after admission. Smoking status was collected through EPR and categorized into never, former or current smoker. Recent hospitalization was defined as hospitalization within 3 months before. IgE mediated penicillin allergy was defined as: itching, angioedema, urticaria, hypotension, anaphylaxis within 1–72 h.

Treatment-related variables were collected from the EPR (maintenance medication or treatment before hospitalization) or hospital pharmacy data (treatment during hospitalization). Recent or frequent antibiotics was defined as antibiotics within 3 months before or more than 4 courses during the preceding year, respectively.

Furthermore, laboratory test results on admission were collected. More information about the laboratory tests can be found elsewhere [20]. Due to the high percentage of missing values of white blood cell differentiation on admission, it was supplemented with data from other time points.

### Statistical analysis

Data analyses were performed using IBM SPSS Statistics® version 25. *P* values < 0.05 were considered significant. In order to examine baseline characteristics, descriptive statistics were applied. Continuous data were described as mean with standard deviation (SD) if normally distributed and as median with interquartile range (Q1-Q3) if not. Categorical variables were described as counts (n) with percentages. An independent samples T-test, Mann–Whitney test, X^2^-test or Fisher’s Exact test were used to test differences in characteristics between AECOPD patients treated with or without antibiotics, respectively. Among antibiotic-treated patients, subgroup analyses were performed to test the differences in characteristics between AECOPD patients initially treated with the first choice amoxicillin-clavulanic acid or another antibiotic. Logistic regression analyses identified factors associated with in-hospital antibiotic use. A manual forward selection process was used to add factors with a *p* < 0.15.

We constructed cumulative survival curves using the Kaplan–Meier method and used Log rank test to identify significant differences. Cox proportional hazards regression analyses were used to compare time to discharge alive and time to in-hospital death between antibiotic-treated patients and non-antibiotic-treated patients. The proportional hazard assumption was checked graphically. For time to discharge alive as outcome, patients were censored at date of in-hospital death or transfer to another hospital. An HR of less than one indicates that the probability of hospital discharge was reduced and thus the LOS was longer. The following covariables were considered as potential confounders: age [21], sex, BMI, FEV_1_, GOLD stage, sputum purulence, pH, smoking status, respiratory failure, recent hospitalization, maintenance theophylline and in-hospital systemic corticosteroid (SCS) use [22]. Model 1 was adjusted for age, model 2 was adjusted for covariates which changed the point estimate by more than 5%, and model 3 was additionally adjusted for FEV_1_. Sensitivity analyses were performed 1) after exclusion of antibiotic users before admission since they could initially be classified as no in-hospital antibiotic users, and 2) after exclusion of late initiated antibiotic users (i.e. from day 2) to verify the robustness of the results. Subgroup analyses were performed 1) for the mainly used antibiotics (see 2.4), and 2) whether or not in-hospital SCS were used. Propensity scores (PS) were calculated. Variables related to in-hospital antibiotic use and LOS (age, BMI and FEV_1_) were included in the PS. Patients were matched with a match tolerance of 0.2.

For in-hospital death as outcome, patients were censored at date of discharge alive. The following covariables were considered as potential confounders for in-hospital death: age, sex, BMI, FEV_1_, comorbidities, pH, CRP, respiratory failure, sputum purulence and in-hospital SCS use [23]. Model 1 was adjusted for age, model 2 was adjusted for covariates which changed the point estimate by more than 5%, and model 3 was additionally adjusted for FEV_1_.

## Results

### Study population

**Fig. 1:**
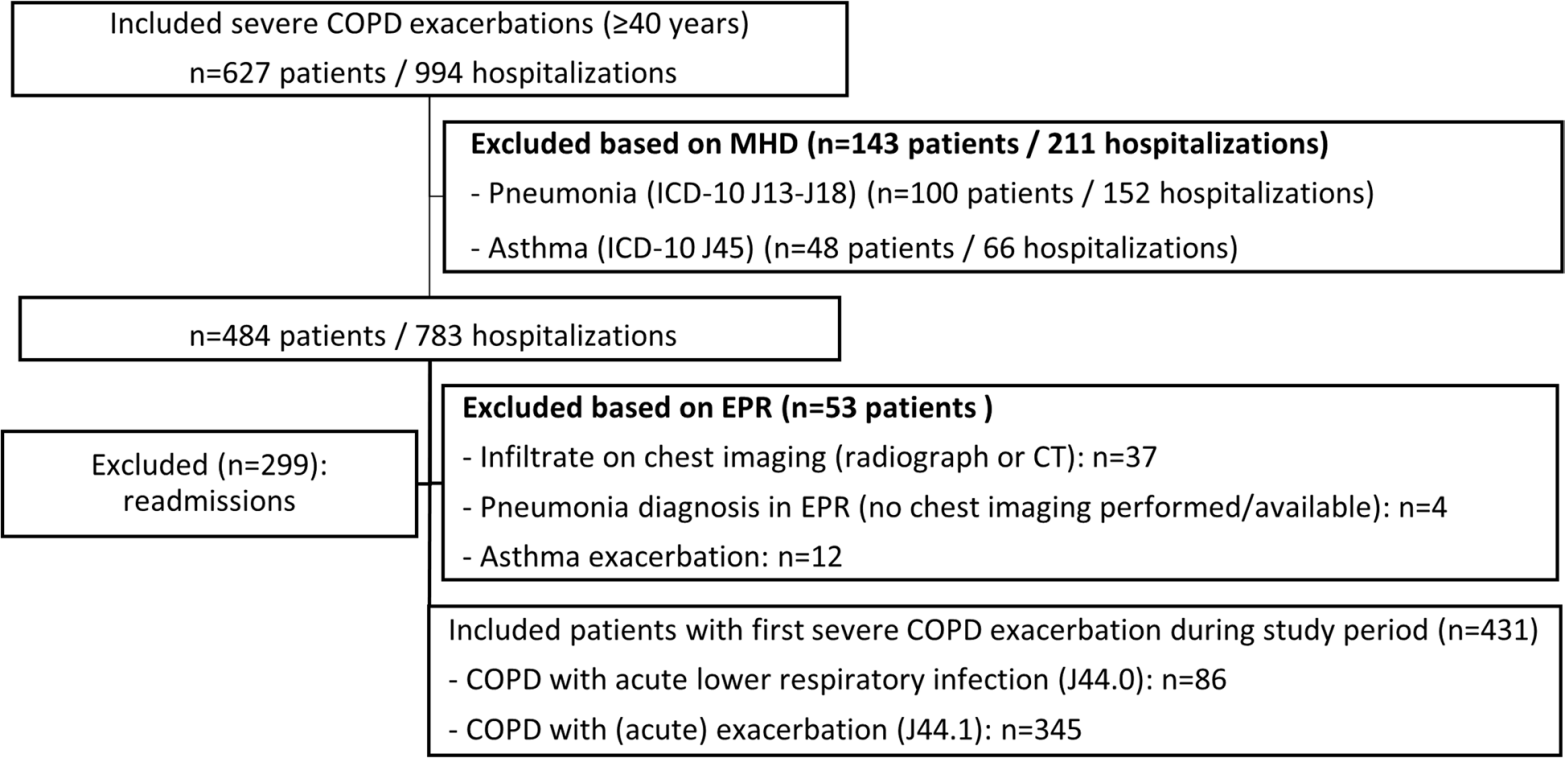
Flow diagram of study population. Abbreviations: COPD, chronic obstructive pulmonary disease; CT, computed tomography; ICD-10, International Classification of Diseases, 10^th^ Revision; MHD, Minimal Hospital Data; EPR, electronic patient records

The study population is represented in Fig. 1. In total, 627 patients were discharged at least once between Jan. 1^st^, 2016 and Jan. 1^st^, 2022. Of these patients, 143 were excluded based on MHD and 53 on EPR. Clinical characteristics were collected for 431 patients during index hospitalization of which eight out of ten patients had COPD with acute exacerbation as verified admission diagnosis. The majority were hospitalized at the regular ward (*n* = 384, 89%). Only 47 patients (11%) were hospitalized at the ICU, of which 39 on the admission day.

### Prevalence of in-hospital antibiotic use

Of all 994 hospitalizations (see Fig. 1), more than half were discharged during winter months (Oct.-March, *n* = 546, 55%). During the index hospitalization, more than two-thirds of patients were treated with antibiotics (*n* = 293/431, 68%). The proportion of patients receiving antibiotics discharged in the summer months (April-Sept., 61%) was lower compared to patients discharged in winter months (Oct.-March, 73%, Chi^2^, *p* = 0.009). This was mainly driven by a lower prevalence in July-Sept. (*n* = 51/89, 57%) compared to Jan.-March (*n* = 115/149, 77%). Patients discharged in April-June (OR 0.55, 95% CI 0.31 to 0.96) and mainly in July-Sept. (OR 0.40, 95% CI 0.22 to 0.70) were less likely to receive antibiotic treatment compared to patients discharged in Jan.-March. Patients discharged in 2021 were less likely to receive antibiotic treatment (OR 0.45, 95% CI 0.23 to 0.88) compared to patients discharged in 2016 after adjustment for discharge quarter.

**Fig. 2:**
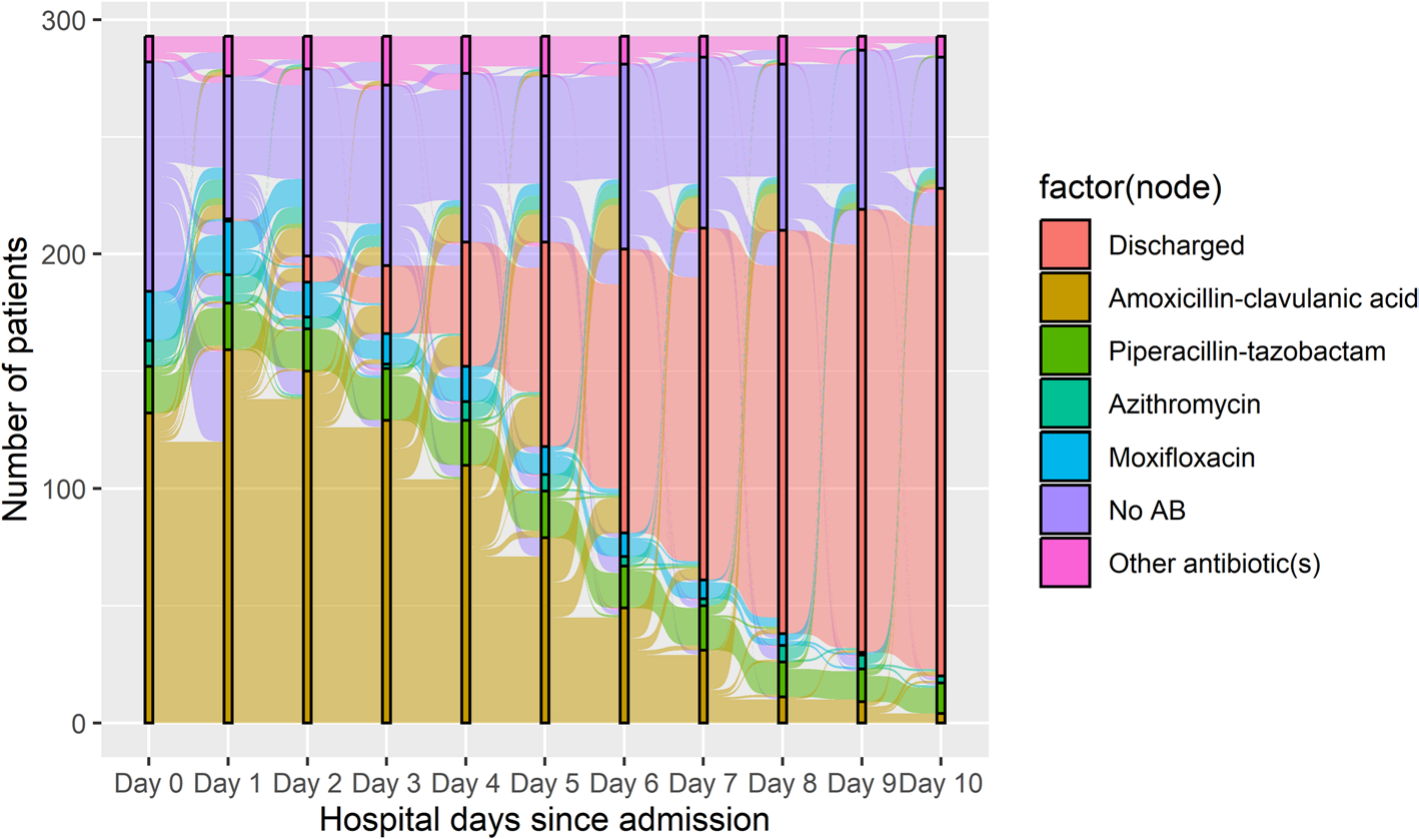
Alluvial plot showing the antibiotic treatment patterns of treated patients (*n* = 293) from one antibiotic to another from admission (= day 0) to discharge or until day 10. The height of each node represents the number of patients receiving the specified treatment (e.g. the majority started on amoxicillin-clavulanic acid and remained until discharge)

The majority of antibiotic-treated patients initially received empirical therapy (*n* = 246, 84.0%) (Table S1). Median (Q1-Q3) antibiotic consumption was 5.50 (3.38–8.20) DDAs per hospitalization (Table S1). Figure 2 illustrates the antibiotic treatment pattern per day during hospitalization of AECOPD patients with at least one antibiotic dispensing (*n* = 293). The majority of patients received amoxicillin-clavulanic acid w/wo other (J01CR02) as initial antibiotic therapy of which 132 on the admission day (= day 0, 45.1%). The following antibiotics were dispensed approximately equally on the admission day: piperacillin-tazobactam w/wo other (J01CR05, *n* = 20, 6.8%) and moxifloxacin w/wo other (J01MA14, *n* = 21, 7.2%). A minority got azithromycin w/wo other on the admission day (J01FA10, *n* = 11, 3.8%). Independent of dispensing day, 188 patients (64.2%) received amoxicillin-clavulanic acid as initial antibiotic therapy.

### Baseline characteristics of included patients

In total, 431 AECOPD patients were included. Of these patients, the majority were men (*n* = 273, 63%) and the mean age at index hospitalization was 70 ± 11 years. Nine out of ten patients were transferred from home. The majority of the antibiotic-treated patients received an antibiotic on the day of admission (*n* = 196, 67%) or the day after admission (*n* = 61, 21%).

Baseline characteristics of patients treated with or without antibiotics are presented in Table 1. AECOPD patients using in-hospital antibiotics differed from patients not using in-hospital antibiotics regarding patient-related variables (age, BMI, diabetes mellitus, cancer and chronic kidney disease), clinical variables (FEV_1_% predicted, sputum volume and purulence, body temperature, recent hospitalization and bronchiectasis), treatment-related variables (maintenance inhaled corticosteroids (ICS), theophylline and azithromycin) and laboratory results (CRP levels, peripheral blood leucocyte, neutrophil and eosinophil counts, and arterial blood pH). Remarkably, 17 COPD patients were neither treated with antibiotics nor SCS, but only with bronchodilators during hospitalization. Seven of them were treated with antibiotics or SCS before admission, three were newly diagnosed COPD, and two had a do not resuscitate code (ICD Z66) ≥ 2.

**Table 1:** Baseline characteristics of untreated and antibiotic (AB) treated AECOPD patients during index hospitalization

Characteristics	Total (*n* = 431)	No in-hospital AB (*n* = 138)	In-hospital AB (*n* = 293)	*P*-value
**Patient-related variables**				
Age in years, mean (SD)	70 (11)	67 (11)	71 (10)	**0.003**
Female sex, n (%)	158 (36.7)	58 (42.0)	100 (34.1)	0.112
BMI in kg/m^2^, median (Q1-Q3)	24.2 (20.5–28.8)	25.3 (21.4–30.1)	23.9 (20.0–28.1)	**0.007**
Diabetes mellitus, n (%)	103 (23.9)	42 (30.4)	61 (20.8)	**0.029**
Cancer, n (%)	65 (15.1)	11 (8.0)	54 (18.4)	**0.005**
Heart failure, n (%)	53 (12.3)	18 (13.0)	35 (11.9)	0.746
Chronic kidney disease, n (%)	64 (14.8)	12 (8.7)	52 (17.7)	**0.014**
Respiratory failure (J96)	72 (16.7)	23 (16.7)	49 (16.7)	0.988
**Clinical variables**				
Stable FEV_1_% pred, median (Q1-Q3)	55 (41–68)	60 (45–73)	52 (39–66)	**0.017**
GOLD I (≥ 80%), n (%)	34 (7.9)	13 (9.4)	21 (7.2)	0.300
GOLD II (50–79%), n (%)	143 (33.2)	48 (34.8)	95 (32.4)	
GOLD III (30–49%), n (%)	106 (24.6)	27 (19.6)	79 (27.0)	
GOLD IV (< 30%), n (%)	46 (10.7)	12 (8.7)	34 (11.6)	
*Anthonisen criteria on admission, n (%)*				
Increased dyspnea	377 (88.5)	120 (89.6)	257 (88.0)	0.644
Increased sputum volume	130 (30.5)	24 (17.9)	106 (36.3)	** < 0.001**
Increased sputum purulence	104 (24.4)	18 (13.4)	86 (29.5)	** < 0.001**
*Anthonisen Type, n (%)*				** < 0.001**
Type 3 (1/3)	255 (59.9)	101 (75.4)	154 (52.7)	
Type 2 (2/3) without sputum purulence	54 (12.7)	12 (9.0)	42 (14.4)	
Type 2 (2/3) with sputum purulence	43 (10.1)	8 (6.0)	35 (12.0)	
Type 1 (3/3)	54 (12.7)	7 (5.2)	47 (16.1)	
Body temperature in °C, median (Q1-Q3)	36.8 (36.4–37.3)	36.6 (36.4–36.9)	36.9 (36.6–37.6)	** < 0.001**
Never smoker, n (%)	8 (2.1)	2 (1.7)	6 (2.4)	0.249
Former smoker, n (%)	193 (51.3)	55 (45.5)	138 (54.1)	
Current smoker, n (%)	175 (46.5)	64 (52.9)	111 (43.5)	
Recent hospitalization, n (%)	51 (11.9)	10 (7.4)	41 (14.1)	**0.046**
Bronchiectasis, n (%)	40 (9.3)	2 (1.5)	38 (13.0)	** < 0.001**
Asthma-COPD overlap, n (%)	33 (7.9)	13 (9.8)	20 (7.0)	0.325
**Treatment-related variables**				
Maintenance inhaled corticosteroids, n (%)	270 (63.7)	76 (55.9)	194 (67.4)	**0.022**
Maintenance theophylline, n (%)	20 (4.7)	2 (1.5)	18 (6.3)	**0.031**
Azithromycin MWF, n (%)	53 (12.5)	3 (2.2)	50 (17.4)	** < 0.001**
Antibiotics before admission, n (%)	117 (27.1)	31 (22.8)	86 (29.8)	0.134
In-hospital systemic corticosteroids, n (%)	362 (84.0)	121 (87.7)	241 (82.3)	0.152
Intensive care unit, n (%)	47 (10.9)	11 (8.0)	36 (12.3)	0.180
No need for ventilatory support, n (%)	93 (21.7)	35 (25.5)	58 (19.9)	0.464
Supplemental oxygen, n (%)	288 (67.1)	90 (65.7)	198 (67.8)	
Non-invasive ventilation, n (%)	44 (10.3)	11 (8.0)	33 (11.3)	
Invasive ventilation, n (%)	4 (0.9)	1 (0.7)	3 (1.0)	
**Laboratory test results**				
CRP in mg/L, median (Q1-Q3)	21.8 (6.5–61.9)	6.7 (2.7–16.1)	33.8 (12.5–82.4)	** < 0.001**
Leucocytes in 10^3^/µL, median (Q1-Q3)	10.19 (7.78–13.34)	9.46 (7.53–11.78)	10.74 (7.97–14.03)	**0.003**
Neutrophil count in /µL, median (Q1-Q3)	6210 (4500–8880)	5680 (4192–8164)	6570 (4580–9115)	**0.038**
Eosinophil count in /µL, median (Q1-Q3)	110 (30–240)	145 (60–303)	100 (21–215)	**0.005**
pH, median (Q1-Q3)	7.417 (7.369–7.452)	7.405 (7.349–7.440)	7.421 (7.374–7.455)	**0.010**
PaCO_2_ in mmHg, median (Q1-Q3)	40.8 (35.2–48.5)	40.4 (35.6–48.9)	41.0 (34.9–48.3)	0.790
PaO_2_ in mmHg, median (Q1-Q3)	63.4 (53.1–73.1)	63.6 (53.1–71.8)	63.4 (53.0–74.2)	0.763
HCO_3_^−^ in mmol/L, median (Q1-Q3)	25.4 (23.0–28.8)	25.0 (22.7–28.7)	25.8 (23.3–29.1)	0.152

*Abbreviations*: *BMI* body mass index, *FEV*_*1*_ forced expiratory volume in 1 s, *GOLD* Global Initiative for Chronic Obstructive Lung Disease, *COPD* chronic obstructive pulmonary disease, *MWF* Monday, Wednesday, and Friday, *CRP* C-reactive protein, *SD* standard deviation

The numbers of the missing values are not shown in this table, but are as follows: BMI: 41 (9.5%), stable FEV_1_: 195 (45.2%), GOLD stage: 102 (23.7%), Anthonisen criteria: 5 (1.2%), body temperature: 16 (3.7%), smoking status: 55 (12.8%), recent hospitalization: 4 (0.9%), bronchiectasis: 3 (0.7%), Ashma-COPD overlap: 12 (2.8%); inhaled corticosteroids: 7 (1.6%), theophylline: 8 (1.9%), azithromycin: 7 (1.6%), antibiotic therapy before admission: 6 (1.4%), CRP: 13 (3.0%), leucocytes: 14 (3.2%), eosinophil count: 76 (17.6%), neutrophil count: 76 (17.6%), pH: 39 (9.0%), PaCO_2_: 38 (8.8%), PaO_2_: 40 (9.3%); HCO_3_^−^: 39 (9.0%)

Baseline characteristics of patients initially treated with amoxicillin-clavulanic acid or another antibiotic are presented in Table S1. Deviation from the first choice amoxicillin-clavulanic acid was significantly associated with clinical variables and treatment-related variables. Regarding clinical variables, the proportion of patients with a penicillin allergy label, recent hospitalization, prior *Pseudomonas aeruginosa* isolation and no empiric treatment for AECOPD were significantly higher in patients treated with another antibiotic. Regarding treatment-related variables, the use of ICS and antibiotics before admission (mainly amoxicillin-clavulanic acid) were significantly associated with the use of another antibiotic than amoxicillin-clavulanic acid.

### Determinants of in-hospital antibiotic use and choice

The results of the univariable and multivariable regression analysis tabulating the determinants of in-hospital antibiotic use and amoxicillin-clavulanic acid as initial therapy are summarized in Table 2 and Table S2, respectively. CRP level was the strongest determinant for in-hospital antibiotic use. Results from the multivariable logistic regression model showed that several patient-related variables (age, BMI, cancer), treatment-related variables (maintenance azithromycin, theophylline), clinical variables (sputum volume and body temperature) and laboratory results (CRP levels) were associated with in-hospital antibiotic use independent of sputum purulence, neutrophil counts, ICS and ICU. No empiric AECOPD treatment was the strongest determinant for deviation from amoxicillin-clavulanic acid.

**Table 2:** Univariable and multivariable regression analysis of the determinants of in-hospital antibiotic use

Variable	Univariable		Multivariable^a^ (*n* = 309)	
	OR [95% C.I.]	*p*-value	aOR [95% CI]	*p*-value
CRP in mg/L	**1.04 [1.03; 1.05]**	** < 0.001**	**1.04 [1.02; 1.06]**	** < 0.001**
Azithromycin MWF	**9.31 [2.85; 30.44]**	** < 0.001**	**12.11 [2.50; 58.61]**	**0.002**
Body temperature in °C	**3.05 [2.10; 4.43]**	** < 0.001**	**5.13 [2.46; 10.70]**	** < 0.001**
Sputum volume	**2.61 [1.58; 4.31]**	** < 0.001**	**2.87 [1.24; 6.66]**	**0.014**
BMI in kg/m^2^	**0.95 [0.92; 0.98]**	**0.002**	**0.94 [0.89; 1.00]**	**0.043**
Age in years	**1.03 [1.01; 1.05]**	**0.003**	**1.04 [1.00; 1.08]**	**0.030**
Maintenance theophylline	**4.43 [1.01; 19.39]**	**0.048**	**7.05 [1.25; 39.83]**	**0.027**
Sputum purulence	**2.69 [1.54; 4.69]**	** < 0.001**	2.34 [0.96; 5.71]	0.061
Neutrophils count in /µL	**1.00009 [1.00002; 1.00016]**	**0.008**	1.00011 [0.99999; 1.00022]	0.064
Cancer	**2.61 [1.32; 5.17]**	**0.006**	**2.60 [1.06; 6.40]**	**0.038**
Sex	0.71 [0.47; 1.08]	0.113	0.54 [0.26; 1.12]	0.100
Inhaled corticosteroids	**1.63 [1.07; 2.48]**	**0.022**	1.75 [0.86; 3.55]	0.123
Intensive care unit	1.62 [0.80; 3.28]	0.183	2.76 [0.71; 10.65]	0.141

Significant estimates (p < 0.05) are indicated in bold

The variables studied for determining in-hospital antibiotic use were: age, sex, BMI, diabetes mellitus, cancer, heart failure, chronic kidney disease, respiratory failure, forced expiratory volume in 1 s % predicted, dyspnea, sputum volume, sputum purulence, body temperature, smoking, recent hospitalization, bronchiectasis, inhaled corticosteroids, theophylline, azithromycin, antibiotics before admission, in-hospital oral corticosteroids, intensive care unit, need for ventilatory support, CRP, leucocytes, neutrophils, eosinophils and pH

*Abbreviations*: *BMI* body mass index, *MWF* Monday, Wednesday, and Friday, *CRP* C-reactive protein

^a^Nagelkerke R^2^: 0.572; Hosmer and Lemeshow goodness-of-fit test *p* value: 0.987; correctly classified: 83.8%

### Outcomes

**Fig. 3:**
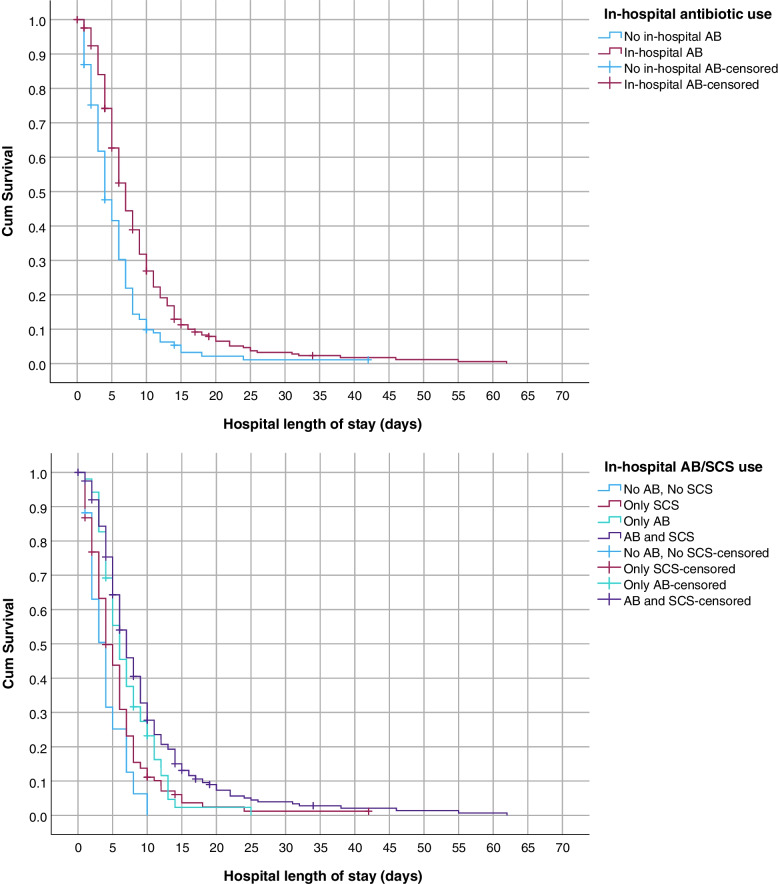
Kaplan–Meier curve of time to discharge according to in-hospital antibiotic use. **A** Main analysis **B** Subgroup analysis 2. *Abbreviations: AB, antibiotics; SCS, systemic corticosteroids*

In total, the median (Q1-Q3) hospital LOS was 6 days (3–9). As shown in Fig. 3, the median hospital LOS was significantly longer in patients treated with antibiotics (6 days [4-10]) compared to patients not treated with antibiotics (4 days [2-7]) (*p* < 0.001, Log rank test). The age-adjusted hazard on discharge was 40% lower for antibiotic-treated patients (Table 3). The results did not change meaningfully after adjusting for possible confounders (BMI, in-hospital SCS use, sputum purulence and FEV_1_), after propensity score (including age, BMI and FEV_1_) adjustment (*n* = 126, HR: 0.63; 95% CI 0.43; 0.92) or after exclusion of patients late initiated antibiotic treatment (i.e. from day 2) (Table S3, sensitivity analysis 2). The sensitivity analysis after exclusion of antibiotic users before admission, resulted in a weaker association with time to discharge (model 2: HR 0.66; 95% CI 0.50; 0.87), and was no longer significant in model 3 (Table S3).

**Table 3:** Cox regression analysis on the association between in-hospital antibiotic use and time to discharge alive

	**Model 1**		**Model 2**	**Model 3**
	**Total**	**HR [95% C.I.], *p*-value**	**HR [95% C.I.], *p*-value**	**HR [95% C.I.], *p*-value**
Main analysis		n events/total = 397/430	n events/total = 362/385	n events/total = 209/228
No in-hospital AB	138	Reference	Reference	Reference
In-hospital AB (all)	293	**0.61 [0.49; 0.75], *p* < 0.001**	**0.60 [0.47; 0.76], *p* < 0.001**	**0.60 [0.43; 0.84], *p* = 0.003**
Subgroup analysis 1: by AB		n events/total = 267/292	n events/total = 249/266	n events/total = 154/168
Co-amoxiclav	188	Reference	Reference	Reference
Pip-tazo	24	**0.54 [0.34; 0.86], *p* = 0.010**	**0.49 [0.30; 0.80], *p* = 0.004**	**0.39 [0.22; 0.69], *p* = 0.001**
Azithromycin	24	1.06 [0.69; 1.65], *p* = 0.782	1.22 [0.78; 1.91], *p* = 0.387	1.09 [0.62; 1.89], *p* = 0.769
Moxifloxacin	32	0.74 [0.50; 1.10], *p* = 0.142	0.82 [0.54; 1.26], *p* = 0.363	0.87 [0.50; 1.52], *p* = 0.628
Other	25	**0.53 [0.34; 0.82], *p* = 0.005**	**0.49 [0.31; 0.79], *p* = 0.003**	**0.45 [0.24; 0.83], *p* = 0.011**
Subgroup analysis 2: by SCS		n events/total = 397/430	n events/total = 362/385	n events/total = 209/228
No AB, no SCS	17	Reference	Reference	Reference
Only SCS	121	**0.56 [0.33; 0.96], *p* = 0.033**	0.60 [0.34; 1.06], *p* = 0.080	0.70 [0.33; 1.49], *p* = 0.349
Only AB	52	**0.50 [0.28; 0.88], *p* = 0.016**	**0.50 [0.27; 0.93], *p* = 0.029**	0.60 [0.27; 1.36], *p* = 0.222
AB and SCS	241	**0.34 [0.20; 0.57], *p* < 0.001**	**0.37 [0.21; 0.65], *p* < 0.001**	**0.42 [0.20; 0.88], *p* = 0.022**

Significant estimates (*p* < 0.05) are indicated in **bold**

*Abbreviations*: *AB* antibiotics, *Co-amoxiclav* amoxicillin-clavulanic acid, *CI* confidence interval, *HR* hazard ratio, *SCS* systemic corticosteroids, *Pip-tazo* piperacillin-tazobactam

Model 1 is adjusted for age (changed point estimate by more than 10%)

Model 2 is additionally adjusted for sputum purulence, body mass index and in-hospital systemic corticosteroid use (H02) (not for subgroup analysis by H02 use) (changed point estimate by more than 5%)

Model 3 is additionally adjusted for forced expiratory volume in 1 s % predicted (changed point estimate by more than 5%)

The results of the subgroupanalyses are presented in Table 3. Subgroup analyses by initial in-hospital antibiotic indicated that the hazard on discharge was significantly lower for patients treated with piperacillin-tazobactam or other antibiotics compared to amoxicillin-clavulanic acid. Subgroup analyses by in-hospital SCS use indicated that the hazard on discharge was significantly lower for all the three groups in model 1, but only for the subgroups receiving antibiotics with or without SCS in model 2 (Fig. 3B and Table 3).

In total, 25/431 AECOPD patients (5.8%) died during index hospitalization. The observed in-hospital mortality was 6.1% among those treated with antibiotics compared with 5.1% among those not treated with antibiotics. In-hospital antibiotic use was not significantly associated with in-hospital mortality (Table 4). Results of the sensitivity analyses are presented in Table S4.

**Table 4:** Cox regression analysis of antibiotic treatment on risk of in-hospital mortality

	**Total**	**Model 1** **HR [95% CI], *p*-value**	**Model 2** **HR [95% CI], *p*-value**	**Model 3** **HR [95% CI], *p*-value**
Main analysis		n events/total = 25/431	n events/total = 18/354	n events/total = 16/208
No in-hospital AB	138	Reference	Reference	Reference
In-hospital AB (all)	293	0.72 [0.30; 1.75], *p* = 0.470	0.64 [0.19; 2.17], *p* = 0.474	0.34 [0.08; 1.45], *p* = 0.145

*Abbreviations*: *AB* antibiotics, *CI* confidence interval, *HR* hazard ratio

Model 1 is adjusted for age

Model 2 is additionally adjusted for C-reactive protein, chronic kidney disease, body mass index, diabetes, pH, cancer, heart failure, sputum purulence and respiratory failure

Model 3 is additionally adjusted for forced expiratory volume in 1 s % predicted

## Discussion

### Prevalence of in-hospital antibiotic use

This retrospective study observed that more than two-thirds (68%) of AECOPD patients were treated with antibiotics during index hospitalization. The observed prevalence of in-hospital antibiotic use (68%) is higher than the expected prevalence of 50% bacterially triggered AECOPD according to available literature [4]. In contrast, this antibiotic prevalence was lower in comparison to 86% (75.6% in Belgium) of admissions treated with an antibiotic according to a European COPD audit between 2010 and 2011 [16]. However, the inclusion of the patients in the European COPD audit was during winter months some years ago [24], and in our study we also observed a higher proportion of patients receiving antibiotics at the beginning of the study and in the winter months (73%). Whether this seasonal variation indicates that proportionally more virally triggered infections are treated with antibiotics [25], or that viral pathogens trigger secondary bacterial infections due to a shift in the microbiome should be further investigated, because respiratory multiplex polymerase chain reaction (PCR) or 16S microbiome analyses were not performed in these patients.

In our study, more than half of the patients received amoxicillin-clavulanic acid as initial therapy which is the preferred antibiotic according to the GOLD guidelines [1], and the Belgian Infectiology Guide [10]. More specifically, amoxicillin-clavulanic acid is recommended for mild or moderate COPD, or severe to very severe COPD patients without risk factors for infection by *Pseudomonas aeruginosa* if initial intravenous treatment is necessary [10]. The following antibiotics were dispensed approximately equally as initial therapy: piperacillin-tazobactam and moxifloxacin. The prevalence of piperacillin-tazobactam use (8.2%) was above the expected prevalence of *P. aeruginosa* in unselected outpatients (4%), but in line with the expected prevalence in COPD patients with advanced airflow obstruction (8–13%) [26]. The prevalence of moxifloxacin use (10.9%) is in line with the prevalence of reported penicillin allergy, but higher than the prevalence of truly allergic patients [27].

### Determinants of in-hospital antibiotic use and choice

In-hospital antibiotic use was determined by characteristics for which antibiotics are recommended by the guidelines or literature. The airflow limitation was more severe (indicated by lower FEV_1_) in patients treated with antibiotics. However, in almost half of patients, no spirometric values were available, which was comparable to the European COPD audit [16]. Moreover, the univariable association with FEV_1_ was no longer significant after inclusion of azithromycin maintenance treatment suggesting that maintenance treatment could be used as a proxy for the underlying COPD severity. Sputum purulence and sputum volume were associated with in-hospital antibiotic use, while no association was observed with dyspnea. This finding may indicate that sputum volume is used as an indicator to start antibiotics, although sputum purulence is the strongest predictor of bacterial infection among the Anthonisen criteria [15]. CRP level was the strongest determinant of in-hospital antibiotic use for which there is moderate evidence to differentiate bacterial AECOPD [11]. Moreover, patients treated with an antibiotic had a significantly lower peripheral blood eosinophil and higher neutrophil count. Previous studies have showed that the airway microbiome differs between neutrophilic and eosinophilic COPD patients [28]. However, the univariable associations with eosinophils and blood leucocyte counts in our study disappeared after including CRP level in the multivariable model, which was supported by the findings of a meta-analysis to summarize biomarkers reporting that blood leucocyte count is not a useful biomarker [11].

In-hospital antibiotic use was also determined by patient-related variables, although not included in the Belgian guidelines. Patients treated with an antibiotic were significantly older and had a lower BMI. Higher age was previously described as a predictor for antibiotic prescribing in general practice [14], and was associated with antibiotic prescription in the European COPD audit [16]. Older age is also a factor taken into account in the guideline of the Dutch Association of Physicians in Chest Medicine and Tuberculosis (NVALT) to start antibiotics more quickly in hospitalized COPD patients [29]. More recently, a subgroup analysis of a meta-analysis observed that the prevalence of bacterial infection in COPD patients increased over age and was lower in studies with a higher proportion of males [4]. However, we did not observe an association with sex which was consistent with Llor and colleagues in general practice [14]. A possible explanation of the observed association with lower BMI is that patients with a BMI ≤ 22 kg/m^2^ were more likely to yield bacterial isolates in sputum in a Greek prospective observational study [30].

Despite the recommendation of antibiotic treatment for patients with acute and chronic respiratory failure in the Belgium Infectiology Guide [10], or requiring mechanical ventilation according to the GOLD 2022 guidelines [1], we observed no significant association between in-hospital antibiotic use and respiratory failure defined as an ICD-10 code for respiratory failure (J96), the need for ventilatory support or hospitalization on ICU. A strong beneficial effect for patients admitted to the ICU needing mechanical ventilation is evidenced by a statistically significant effect on mortality [6]. However, this was based on only one randomized controlled trial, performed more than 20 years ago in which patients did not receive corticosteroids [6, 31].

Antibiotic choice was determined by guideline recommended indications to deviate from the first choice antibiotic amoxicillin-clavulanic acid (no empirical AECOPD treatment, penicillin allergy and recent hospitalization). The risk factors for infection by *Pseudomonas aeruginosa* for which we observed a significant univariable association were recent hospitalization, recent antibiotics and prior *Pseudomonas aeruginosa* isolation of which only recent hospitalization was included in the multivariable model. Patients with a penicillin allergy label were less likely to be treated with amoxicillin-clavulanc acid. To avoid overuse of second-line treatment, it is important that patients with an IgE penicillin allergy are correctly labeled. In 2017, 6.1% of patients reported an antibiotic allergy (of which 90% penicillin) at Ghent University Hospital surgical day hospitalization [32]. However, based on a prospective observational study in a Belgian outpatient population, 91% of the participants could be safely delabeled [33].

Antibiotic choice was also determined by antibiotic use before admission and azithromycin maintenance treatment which are not included in guidelines. Antibiotic use before admission was significantly associated with dispensing of another antibiotic than amoxicillin-clavulanic acid which could be due to clinical failure on initiated amoxicillin-clavulanic treatment by the general practitioner. The association between antibiotic prescription before and during admission observed in the European COPD audit was only a trend in our study suggesting that the need to continue antibiotics is questioned [16]. Therefore, Antimicrobial Stewardship interventions should not only target in-hospital antibiotic use, but also antibiotic use in primary care. Azithromycin maintenance therapy was not only a determinant of in-hospital antibiotic use, but also of another antibiotic than amoxicillin-clavulanic acid. This could be due to continuation of the chronic immunomodulatory treatment in hospital [1, 34] or by the more severe underlying COPD.

We focused on demographic, clinical and treatment-related variables and laboratory test results at the individual patient level. However, we could not exclude that variables related to the prescriber, the hospital and the policy level might also have influenced the in-hospital antibiotic use or choice.

### Outcomes

The median LOS in our study (6 days) was 1 day shorter compared to the median LOS (7 days) in a European COPD audit performed in 2010–2011 [22]. In our study, we observed that the LOS was longer for patients receiving antibiotic therapy. This finding was consistent with this European COPD audit which observed that antibiotic use was strongly associated with an increased risk of a LOS longer than the median [22]. LOS was similar between the antibiotic and placebo group in a meta-analysis of four inpatient randomized controlled trials [6]. In contrast, we also observed a longer LOS for patients treated with antibiotics compared to patients not receiving antibiotics nor corticosteroids in the subgroup analysis. Another American retrospective study observed no significant differences in hospital LOS between the GOLD guideline-recommended or inappropriate antibiotic therapy [18]. Subgroup analyses by initial antibiotic indicated that the hazard on discharge was significantly lower for piperacillin-tazobactam and other antibiotics compared to amoxicillin-clavulanic acid. A recent American retrospective, multicenter cohort study observed less treatment failure (initiation of new antibiotic and readmission) and shorter LOS in the azithromycin group compared to the beta-lactam group [35]. However, the most commonly prescribed beta-lactam was ceftriaxone [35], and not amoxicillin-clavulanic acid as in our study. Moreover, azithromycin was probably not used as treatment in our study, but as chronic immunomodulatory treatment [1, 34].

Possible explanations for our findings are a more severe AECOPD, slower response to treatment or ‘harm’ due to antibiotics. First, the association could be due to a more severe AECOPD or patient condition [22]. This hypothesis seems less probable, since (propensity score) adjusted analyses yielded similar effect estimates. However, unmeasured confounders may still be present. Secondly, the association may be due to a slower response to treatment of causative phenotype of AECOPD. Patients with a respiratory infectious phenotype had longer LOS and higher COPD Assessment Test score than non-infectious patients in a previous study [36], and patients with eosinophilic AECOPD had a shorter LOS [37]. It is possible that patients not treated with antibiotics with an eosinophilic exacerbation (suggested by the higher eosinophil count in this group) more rapidly respond to (corticosteroid) treatment. Finally, patients treated with (prolonged) antibiotic therapy could in theory experience (more) adverse effects requiring a longer hospital stay. The Cochrane meta-analysis showed that antibiotic-treated patients had more (but not statistical significant) frequent adverse effects [6]. This explanation seems to contribute only partially, since only a few patients had an ICD-10 code for a possible adverse effect or were switched to another antibiotic because of adverse drug effects [data not shown]. The LOS was not significantly longer in those on antibiotics with an ICD-10 code for a possible adverse effect (*n* = 12; 8.5 days) compared to those on antibiotics without record of a possible adverse effect (*n* = 281, 6 days, Mann–Whitney, *p* = 0.307).

In this study, 5.8% died during index hospitalization compared to the overall short-term cumulative incidence of death of 3.6% (1.8–20.4%) observed in a systematic review of 17 studies [23]. We observed no association between antibiotic therapy and in-hospital mortality. Another large retrospective cohort study throughout the United States observed that antibiotic treatment was associated with a 40% risk reduction of in-hospital mortality [17]. Since patients starting on antibiotics after the third hospital day were grouped with controls who were not treated with antibiotics, results might have favored the (early initiating) antibiotic group because late treatment is likely associated with clinical deterioration [17]. We have grouped late initiating patients to the antibiotics group to avoid immortal time bias as the patients must survive the first two days to receive late antibiotic therapy and did a sensitivity analysis excluding these patients which confirmed no significant association with mortality.

### Strengths and limitations

A first strength of our study is the long study period of 6 years. Other observational studies investigating the association between antibiotic use and outcomes had shorter study periods. Moreover, in addition to the MHD and hospital pharmacy data, we also collected clinical data which led to a very rich dataset.

Our study had some limitations. First, as this study was a real-world observational study, COPD patients were not randomly treated, but upon the clinical judgment of the clinician which could have led to bias by the symptom severity of the AECOPD. Second, the study was a single center study, only involving Ghent University Hospital, a tertiary care center, institution-specific confounders may exist and the results may not be generalizable to all COPD patients with severe acute exacerbations. Third, as this was a retrospective study, this research relied on routinely collected health data. As a result, incomplete medical records or errors in medical records could not be ruled out, which could lead to information bias. For example, white blood cell differentiation was only available on admission in almost half of the cases and was supplemented with results at other time points whereby eosinophil levels may be influenced by SCS therapy. Some potential misclassification of patients with clinically confirmed AECOPD may have occurred by including patients based on J44 ICD-10 codes [38]. However, we tried to limit misclassification by excluding pneumonias and ‘pure' asthma exacerbations. In a sensitivity analysis only excluding patients with a concomitant diagnosis of pneumonia, but not asthma, the antibiotic prevalence was 67% (compared to 68%).

We were not able to investigate long-term outcomes. Readmission would be underestimated in our study due to readmission to other hospitals in the region. Moreover, we could not exclude the possibility that some dispensed antibiotics were prescribed for a co-infection instead of the initial COPD exacerbation. This highlights the need for the inclusion of an indication-based coded field in electronic health records. We aimed to estimate the days of antibiotic treatment as best as possible by using DDAs instead of DDDs. Still, DDAs will overestimate the actual length of antibiotic therapy for patients receiving combination therapy and underestimate the actual length of antibiotic therapy for patient receiving further antibiotic therapy before or after admission.

### Future perspectives

An upcoming randomized, double-blind, placebo-controlled study (ABACOPD) targeting to include 980 patients with moderate AECOPD receiving state-of-the-art treatment will be the first study to investigate whether use of placebo is not inferior to antibiotic treatment [39]. The results of this study may help to further optimize the risk–benefit ratio of antibiotics for patients with a severe COPD exacerbation. However, the first results showed that the study failed to demonstrate non-inferiority of placebo to sultamicillin across all AECOPD patients, but suggests that antibiotic therapy could be withheld in GOLD stage I- II patients [40].

Our study highlights the need for Antibiotic Stewardship and more evidence about “SMART” biomarkers to support clinicians whether or not to prescribe antibiotics for AECOPD. Most of the approaches to define a bacterial AECOPD fail to discriminate between airway colonization and the cause of the infection when detecting bacteria. In the case of co-infections it is even more difficult to differentiate between the relative effect of the isolated pathogens [11]. Future guidelines should guide clinicians not only in the choice of antibiotic, but also the use to increase the benefit-risk ratio and include recommendations regarding laboratory results and patient-related variables.

## Conclusion

In summary, this observational study in a Belgian tertiary hospital demonstrated that several patient-, hospitalization-, and treatment- related variables, clinical variables and laboratory results were associated with in-hospital antibiotic use in severe AECOPD patients. AECOPD patients treated with antibiotics were associated with a longer LOS compared to AECOPD patients not treated with antibiotics, which may be linked to their disease severity, slower response to treatment or ‘harm’ due to antibiotics.

## Supplementary Information

Additional file 1.

## Data Availability

The datasets generated during and/or analyzed during the current study are available from the corresponding author on reasonable request.

## Abbreviations

AB: Antibiotics
AECOPD: Acute exacerbations of COPD
APR-DRG: All Patient Refined Diagnosis Related Groups
ATC: Anatomical Therapeutic Chemical
BAPCOC: Belgian Antibiotic Policy Coordination Committee
BMI: Body mass index
CI: Confidence interval
Co-amoxiclav: Amoxicillin-clavulanic acid
COPD: Chronic obstructive pulmonary disease
CRP: C-reactive protein
CT: Computed tomography
DDA: Defined daily doses adjusted
EPR: Electronic patient records
FEV_1_: Forced expiratory volume in one second
GOLD: Global Initiative for Chronic Obstructive Lung Disease
HR: Hazard ratio
ICD: International Classification of Diseases
ICS: Inhaled corticosteroids
ICU: Intensive care unit
Ig: Immunoglobulin
Jan.: January
LOS: Length of stay
MHD: Minimal Hospital Data
MWF: Monday, Wednesday, and Friday
n: Counts
NVALT: Dutch Association of Physicians in Chest Medicine and Tuberculosis
Oct.: October
Pip-tazo: Piperacillin-tazobactam
PS: Propensity scores
PCR: Polymerase chain reaction
Q1-Q3: Interquartile range
SCS: Systemic corticosteroid
Sept.: September
SD: Standard deviation
w/wo: With/without
