# Supplementary material for: In-hospital antibiotic use for severe chronic obstructive pulmonary disease exacerbations: a retrospective observational study
Source: BMC Pulm Med. 2023 Apr 25;23:138. doi: 10.1186/s12890-023-02426-3 (PMC10127022; doi:10.1186/s12890-023-02426-3)
Supplement: Supplementary file 1 — Additional file 1. [file 12890_2023_2426_MOESM1_ESM.docx]

# **SUPPLEMENT**

**Table S1** Baseline characteristics of patients treated with amoxicillin-clavulanic acid (Co-amoxiclav) or other antibiotics (AB)

| **Characteristics** | **In-hospital AB**  **(n=293)** | **Co-amoxiclav (n=188)** | **Other AB**  **(n=105)** | **P-value** |
| --- | --- | --- | --- | --- |
| *Patient-related variables* | | | | |
| Age in years, mean (SD) | 71 (10) | 70 (10) | 72 (11) | 0.057 |
| Female sex, n (%) | 100 (34.1) | 65 (34.6) | 35 (33.3) | 0.830 |
|  |  |  |  |  |
| *Clinical variables* | | | | |
| Stable FEV_1_% pred, median (Q1-Q3) | 52 (39-66) | 52 (40-67) | 51 (36-65) | 0.940 |
| GOLD I (≥80%), n (%)  GOLD II (50-79%), n (%)  GOLD III (30-49%), n (%)  GOLD IV (<30%), n (%) | 21 (7.2)  95 (32.4)  79 (27.0)  34 (11.6) | 10 (5.3)  69 (36.7)  48 (25.5)  18 (9.6) | 11 (10.5)  26 (24.8)  31 (29.5)  16 (15.2) | 0.092 |
| Penicillin allergy label, n (%)  No  IgE mediated  Unspecified | 245 (89.7)  22 (8.1)  6 (2.2) | 165 (95.9)  6 (3.5)  1 (0.6) | 80 (79.2)  16 (15.8)  5 (5.0) | **<0.001** |
| Recent hospitalization, n (%) | 41 (14.1) | 19 (10.2) | 22 (21.2) | **0.010** |
| Bronchiectasis, n (%) | 38 (13.0) | 20 (10.6) | 18 (17.1) | 0.112 |
| Prior *Pseudomonas* isolation, n (%) | 17 (6.0) | 6 (3.2) | 11 (11.0) | **0.008** |
| Indication initial antibiotic, n (%)  Not specified  Prophylactic  Empirical AECOPD  Targeted AECOPD  Other infection | 2 (0.7)  17 (5.8)  246 (84.0)  16 (5.5)  12 (4.1) | 0 (0.0)  2 (1.1)  175 (93.1)  7 (3.7)  4 (2.1) | 2 (1.9)  15 (14.3)  71 (67.6)  9 (8.6)  8 (7.6) | **<0.001** |
|  |  |  |  |  |
| *Treatment-related variables* | | | | |
| Maintenance inhaled corticosteroids, n (%)  Azithromycin MWF, n (%) | 194 (67.4)  50 (17.4) | 114 (62.0)  18 (9.8) | 80 (76.9)  32 (30.8) | **0.009**  **<0.001** |
| Antibiotics before admission, n (%) | 86 (29.8) | 37 (20.0) | 49 (47.1) | **<0.001** |
| Frequent antibiotics, n (%) | 4 (1.4) | 3 (1.6) | 1 (1.0) | 1.000 |
| Recent antibiotics, n (%) | 103 (35.2) | 48 (25.5) | 55 (52.4) | **<0.001** |
| In-hospital systemic corticosteroids, n (%) | 241 (82.3) | 153 (81.4) | 88 (83.8) | 0.602 |
| DDAs, median (Q1-Q3) | 5.50 (3.38-8.20) | 5.25 (3.25-7.69) | 6.29 (4.00-9.39) | **0.015** |

Abbreviations: SD, standard deviation; FEV_1_, Forced Expiratory Volume in 1 second; GOLD, Global initiative for Chronic Obstructive Lung Disease; IgE, Immunoglobulin E; AECOPD: Acute exacerbation of Chronic Obstructive Pulmonary Disease; MWF, Monday, Wednesday, and Friday; DDAs, Defined Daily Doses adjusted to the Belgian situation in hospitals

The numbers of the missing values are not shown in this table, but are as follows: stable FEV_1_: 121 (41.3%), GOLD stage: 64 (21.8%), penicillin allergy: 20 (6.8%), recent hospitalization: 2 (0.7%), prior *Pseudomonas* isolation: 8 (2.7%), maintenance inhaled corticosteroids: 5 (1.7%), Azithromycin MWF: 5 (1.7%), antibiotics before admission: 4 (1.4%)

**Table S2** Univariable and multivariable regression analysis of the determinant of initial amoxicillin-clavulanic acid

| **Variable** | **Univariable** | | **Multivariable* (n=262)** | |
| --- | --- | --- | --- | --- |
|  | **OR [95% C.I.]** | **p-value** | **aOR [95% CI]** | **p-value** |
| No empiric AECOPD treatment | **0.16 [0.08; 0.31]** | **<0.001** | **0.12 [0.05: 0.27]** | **<0.001** |
| Antibiotics before admission | **0.28 [0.17; 0.48]** | **<0.001** | **0.19 [0.10; 0.37]** | **<0.001** |
| IgE mediated penicillin allergy | **0.18 [0.07; 0.48]** | **<0.001** | **0.13 [0.04: 0.40]** | **0.001** |
| Unspecified penicillin allergy | **0.10 [0.01; 0.84]** | **0.035** | **0.06 [0.01; 0.64]** | **0.019** |
| Azithromycin MWF | **0.24 [0.13; 0.46]** | **<0.001** | **0.38 [0.17; 0.82]** | **0.014** |
| Recent hospitalization | **0.42 [0.22; 0.82]** | **0.011** | 0.49 [0.21; 1.12] | 0.091 |

Significant estimates (p<0.05) are indicated in **bold**.

*Nagelkerke R^2^ : 0.386; Hosmer and Lemeshow goodness-of-fit test p value: 0.334; correctly classified: 76.0%

Abbreviations: AECOPD: acute exacerbation of Chronic Obstructive Pulmonary Disease; IgE: immunoglobulin E; MWF, Monday, Wednesday, and Friday

The variables studied for determining antibiotic choice were: age, sex, forced expiratory volume in 1 second % predicted, Global initiative for Chronic Obstructive Lung Disease stage, penicillin allergy, recent hospitalization, bronchiectasis, prior *Pseudomonas* isolation, indication initial antibiotic, maintenance inhaled corticosteroids, azithromycin MWF, antibiotics before admission, frequent antibiotics, recent antibiotics

**Table S3** Sensitivity analyses: cox regression analysis on the association between antibiotic treatment (AB) and time to discharge

|  |  | **Model 1** | **Model 2** | **Model 3** |
| --- | --- | --- | --- | --- |
|  | **Total** | **HR [95% C.I.], p-value** | **HR [95% C.I.], p-value** | **HR [95% C.I.], p-value** |
| *Sensitivity analysis 1: excl AB before admission* | | *n events/total=288/313* | *n events/total=261/27980* | *n events/total=142/157* |
| No in-hospital AB, no AB before | 107 | Reference | Reference | Reference |
| In-hospital AB, no AB before | 207 | **0.69 [0.54; 0.88], p=0.003** | **0.66 [0.50; 0.87], p=0.003** | 0.74 [0.49; 1.10], p=0.137 |
|  |  |  |  |  |
| *Sensitivity analysis 2: excl late initiated AB* | | *n events/total=362/394* | *n events/total=328/350* | *n events/total=189/207* |
| No in-hospital AB | 138 | Reference | Reference | Reference |
| AB initiated on day 0 or day 1 | 257 | **0.63 [0.51; 0.79], p<0.001** | **0.62 [0.48; 0.79], p<0.001** | **0.64 [0.45; 0.90], p=0.011** |

Abbreviations: AB, antibiotics; HR, Hazard Ratio; CI, Confidence Interval

Model 1 is adjusted for age

Model 2 is additionally adjusted for sputum purulence, body mass index and in-hospital systemic corticosteroid use (H02)

Model 3 is additionally adjusted for forced expiratory volume in 1 second % predicted

**Table S4** Sensitivity analyses: cox regression analysis on the association between antibiotic treatment (AB) and time to in-hospital death

|  |  | **Model 1** | **Model 2** | **Model 3** |
| --- | --- | --- | --- | --- |
|  | **Total** | **HR [95% C.I.], p-value** | **HR [95% C.I.], p-value** | **HR [95% C.I.], p-value** |
| *Sensitivity analysis 1: excl AB before admission* | | *n events/total=20/31* | *n events/total=14/260* | *n events/total=13/145* |
| No in-hospital AB, no AB before | 107 | Reference | Reference | Reference |
| In-hospital AB, no AB before | 207 | 0.66 [0.26; 1.67], p=0.384 | 0.59 [0.15; 2.40], p=0.464 | 0.30 [0.06; 1.60], p=0.158 |
|  |  |  |  |  |
| *Sensitivity analysis 2: excl late initiated AB* | | *n events/total=25/395* | *n events/total=18/321* | *n events/total=16/187* |
| No in-hospital AB | 138 | Reference | Reference | Reference |
| AB initiated on day 0 or day 1 | 257 | 0.83 [0.34; 2.02], p=0.685 | 0.82 [0.24; 2.82], p=0.751 | 0.48 [0.11; 2.13], p=0.337 |

Abbreviations: AB, antibiotics; HR, Hazard Ratio; CI, Confidence Interval

Model 1 is adjusted for age

Model 2 is additionally adjusted for C-reactive protein, chronic kidney disease, body mass index, diabetes, pH, cancer, heart failure, sputum purulence and respiratory failure

Model 3 is additionally adjusted for forced expiratory volume in 1 second % predicted
